# Newborn Care Practices in Urban Slums of Lucknow City, UP

**DOI:** 10.4103/0970-0218.62570

**Published:** 2010-01

**Authors:** Pratibha Gupta, VK Srivastava, Vishwajeet Kumar, Savita Jain, Jamal Masood, Naim Ahmad, JP Srivastava

**Affiliations:** Department of Community Medicine, Era's Lucknow Medical College, Lucknow, India; 1 Department of Upgraded of SPM (Community Medicine), Lucknow, India; 2 Department of KGMU-JHU Collaborative Projects, King George's Medical University, Lucknow, India

**Keywords:** Birth asphyxia, breastfeeding, hypothermia, newborn

## Abstract

**Objectives:**

To study the knowledge and practices related to newborn care in urban slums of Lucknow city, UP, and to identify critical behaviors, practices, and barriers that influence the survival of newborns.

**Materials and Methods:**

A cross-sectional study in urban slums of Lucknow city, UP, included 524 women who had a live birth during last 1 year preceding data collection. Data were analyzed using statistical software SPSS 10.0 for windows.

**Results:**

Study findings showed that about half of the deliveries took place at home. Majority (77.1%) of the mothers believed that baby should be bathed with warm water and dried with clean cloth and 79.7% mothers practiced it. Only 36.6% mothers initiated breast-feeding within 1 h of birth and 30.2% initiated after 1 day. The mothers who have not given colostrum to their baby, in majority the reason was customs.

**Conclusion:**

In majority of cases, correct knowledge and correct practices regarding newborn care were lacking among mothers and this should be promoted through improved coverage with existing health services.

## Introduction

Newborn mortality is one of the world's most neglected health problems. It is estimated that globally four million newborns die before they reach 1 month of age and another four million are stillborn every year. Death during the neonatal period (the first 28 days of life) accounts for almost two-thirds of all deaths in the first year of life and 40% of deaths before the age of five.(1)

The global burden of neonatal death is estimated to be 5.0 million of which 3.2 million deaths occur during the first week of life.(2) Each year, 26 million infants born in India. Of these, nearly 1.2 million die during the neonatal period, before completing 4 weeks of life, amounting to one quarter of all the neonatal deaths in the world.(3) India, thus contributes 30% of the 3.9 million neonatal deaths worldwide.(4) Global under five and infant mortality rates have declined over the past four decades, but high neonatal mortality rates have remained relatively unchanged. The primary causes of neonatal death are believed to be complications of prematurity (28%), sepsis and pneumonia (26%) birth asphyxia and injuries (23%), tetanus (7%), congenital anomalies (7%), and diarrhea (3%), with low birth contributing to a large proportion of deaths.

Since the problem of availability of quality care to all, the neonate is multifaceted, the solution too have to be likewise. There is sufficient evidence to show that most of the basic neonatal care can be delivered at homes through primary care in a highly cost-effective manner. Hence, to reduce neonatal mortality, strategies must be developed for safe home deliveries including essential neonatal care, besides devising means of proper care of the neonate in domestic settings and ensuring proper referral of only those neonates who cannot be managed at home. Many of the life-threatening conditions could be prevented or treated with low technology, improved labor and delivery care, and attention to the physiological needs of the newborn. The causes of neonatal mortality, the organization and coverage of delivery care, resuscitation, low birth weight, hypothermia, low technology warming, reducing infection, etc. are some important areas that have to be addressed.(5)

The present study aims to study the knowledge and practices of families in relation to newborn care viz. prevention of hypothermia, prevention of birth asphyxia, colostrum feeding, early initiation of breast feeding, and pre-lacteal feeding so as to improve neonatal survival and decrease morbidity and mortality in India.

### Objectives

1. To study the knowledge and practices related to newborn care in urban slums of Lucknow city.
2. To identify critical behaviors and barriers that influence the survival of newborns.

## Materials and Methods

### Study population

The present study was carried out among newborns in urban slums of Lucknow city. The actual accessible population from which the sample was taken was defined as “all those households having mothers who gave birth to a live born within the last one year.”

### Period of study

The study was carried out from August 2005 to September 2006 which included the development of study tools, collection of data, analysis, tabulation of findings, and interpretation of results.

### Sampling

Sample size was calculated on the basis of percent distribution of children who were fed colostrum. According to NFHS, UP, (1998-1999)(6) percentage of children who were fed colostrum was 25%. An absolute permissible error of 4% was taken to calculate the sample size. Considering a 10% of non-response, the sample size came out to be 517; however in the present study 524 children were covered.

### Sampling technique

There are eight maternal and child health centre (Bal Mahila Chikitsalaya) in Lucknow city. For sampling purpose at first stage, one maternity center was selected randomly which was Aliganj, Maternal and Child Health Centre. This center covers a total of 33 slums covering a population of about 40,000. Considering an average family size of 5.6 for the slums of Lucknow, there would be about 7142 households and 1100 children below 1 year of age. In order to cover desired sample size of 524, systematic random sampling was used and every second household was surveyed.

### Analysis and interpretation of data

The data were tabulated on Microsoft Excel sheet and analyzed using the software SPSS 10.0 for windows. Discrete data were analyzed using the Chi-square test.

## Results

A total of 524 families were surveyed. There were 29.4% Muslims and 70.6% Hindus families. Amongst Hindus 33.6% belonged to OBC, 26.3% belonged to SC/ST and 10.7% belonged to general caste. The majority (70%) of the families were nuclear type. More than half (59.5%) of the mothers were illiterate. About one-third (33.2%) of the mothers had education level upto junior/high school level [Table 1].

**Table 1:** Socio-demographic characteristics of the study population (N = 524)

Characteristics	No.	Percent
Religion and caste		
Muslim	154	29.4
Hindu	370	70.6
Gen	56	10.7
OBC	176	33.6
SC/ST	138	26.3
Type of family		
Nuclear	367	70.0
Joint	157	30.0
Socio-economic status		
I	0	0.0
II	40	7.6
III	35	6.7
IV	66	12.6
V	351	67.0
Family size		
<5	195	37.2
5-6	192	36.6
>6	137	26.1
Mother's education		
Illiterate	312	59.5
Up to high school	174	33.2
Intermediate and +	38	7.2

The study findings shows that about half (51.7%) of the deliveries took place at home followed by govt. health facility (28.4%). Only 19.8% of the deliveries took place at private health facility. Knowledge and practice about hypothermia and birth asphyxia were asked only in case of home deliveries.

Majority (77.1%) of the mothers believed that baby should be bathed with warm water and dry with a clean cloth and only 15.1% said that baby should not be bathed and only dry up with a clean cloth. To prevent birth asphyxia, 42.4% of the mothers told about hanging upside down and 38% expressed about slapping the back [Table 2].

**Table 2:** Knowledge of mothers about prevention of hypothermia and birth asphyxia (N = 271)

Critical behavior	No.	Percentage
Hypothermia		
Not to be bathed and only dry up with a clean cloth	41	15.1
Should be washed with warm water and dry with a clean cloth	209	77.1
Wrapping/clothing	21	7.8
Birth asphyxia		
Hanging the baby upside down	115	42.4
Slapping the back	103	38.0
Cleaning of oral cavity with the help of finger	27	10.0
Don't know	26	9.6

About one-third (30.2%) of the mothers were of opinion that the initiation of breastfeeding should be within 1 h of birth and 31.6% were of opinion that it should be 1-24 hours of birth. Only 16.2% said about this that it should be after 1 day. Nearly half (49%) of the mothers said that colostrum might prevent from illnesses. However, 39.1% thought that by giving colostrum, baby might become healthy [Table 3].

**Table 3:** Knowledge of mothers about breast feeding practices

Breastfeeding	(N = 524)	
	
	No.	Percentage
Initiation of breast-feeding		
Within 1 h	158	30.2
One to 24 h	166	31.6
After 1 day	85	16.2
Don't know	115	21.9
*Use colostrum		
Prevents illness	257	49.0
Baby becomes healthy	205	39.1
Don't know	104	19.8

* Multiple response

The majority of the newborns (79.7%) were washed with warm water and dried up with a clean cloth immediately after birth, while only 18.1% of newborns were not given a bath and only dried up with a clean cloth. The majority (90.4%) of the newborns cried immediately after birth. Out of those who did not cry immediately after birth, 46.2% of the mothers did not remember the steps taken to make baby cry. However, in 42.3% cases slapping the back and in 11.5% hanging upside down were done [Table 4].

**Table 4:** Practices of mothers regarding prevention of hypothermia and birth asphyxia

Practices	(N =271)	
	
	No.	Percentage
Hypothermia		
Not bathed and completely dried with clean cloth	49	18.1
Washed with warm water and dried with a clean cloth	216	79.7
Wrapping/clothing	6	2.2
Birth asphyxia		
Baby cried immediately		
Yes	245	90.4
No	26	9.6
If no, what steps taken to make baby cry?		
Hanging upside down	3	11.5
Slapping the back	11	42.3
Did not remember	12	46.2

Just more than one-third (36.6%) of the mothers initiated breastfeeding within 1 h of birth and 30.2% initiated after 1 day. There were 33.2% mothers who initiated within 1-24 h. Reasons for late initiation of breastfeeding were family customs and belief in 52.1% newborns. More than one-third (43.5%) mothers gave the colostrum to their baby. However, out of those who did not give colostrum, 66.9% did not give it because they thought that it is harmful for the baby. Similar percentages (14.5%) of mothers did not give colostrum due to ignorance about advantages of this and prohibited by elderly female. Only 4.1% of the mothers did not give colostrum due to the absence of milk secretion [Table 5].

**Table 5:** Practices of mothers regarding breast-feeding

Practices	(N =524)	
	
	No.	Percentage
Initiation of breast-feeding		
Initiation of breastfeeding (*n* =524)		
Within 1 h (early)	192	36.6
1-24 h	174	33.2
After 1 day	158	30.2
Reasons for late initiation (*n* = 332)		
Discomfort to mother	56	16.9
No milk secretion	103	31.0
Family customs/belief	173	52.1
Given colostrum (*n* =524)		
Yes	228	43.5
No	296	56.5
If no, reasons (*n* =296)		
Ignorance about advantages	43	14.5
Prohibited by elderly female	43	14.5
Absence of milk secretion	12	4.1
Harmful for the baby	198	66.9

## Discussion

Improving newborn survival is a major priority in child health today. Specific programs for enhancing the maternal and child health have been in place since the early 1950s till date, like the MCH program, immunization, ORS for the control of diarrheal disease, anemia, and vitamin A prophylaxis program, CSSM, and current RCH II.

The present study showed that the maximum (77.1%) numbers of newborns were given a bath immediately after birth. Similarly, Singh (2002)(7) in a study in rural area of Ghaziabad U.P. also reported that bath was given in 71.2% of the newborns. These findings show that there was very less awareness in community regarding prevention of hypothermia. In the community if they had the information that the bathing immediately after birth causes hypothermia and may lead to death, the practice may have been different. Similarly, Kumar *et al*. (1998)(8) in a study in Haryana found that more than 40% of infants were reportedly not wiped after birth and 65% were bathed within 24 h of delivery.

Another major cause of neonatal mortality is asphyxia, which is usually due to mishandling of the baby at birth. In this study, it was observed that regarding the methods of prevention of birth asphyxia, knowledge of the mother was limited to about hanging the baby upside down in 42.4% and slapping the back immediately after birth in 38% of birth to clean the airway of secretion. In the present study, majority (90.4%) of the newborn cried immediately after birth. In the present study, out of those who did not cry immediately after birth in 42.3% slapping the back and in 11.5% hanging the baby upside down methods were practiced to make the baby cry. However, 46.2% of mothers did not remember the steps taken to make their baby cry.

Similarly, in the study of Singh (2002) in a rural area of Ghaziabad, it was also observed that 541% mothers knew about hanging the baby upside down while 55.9% said slapping the back revived a newborn who was not breathing.

In the present study, it was found that the 69.8% mothers initiated breast-feeding within 24 h. Similarly, Ramkrishna *et* *al*. (2000)(9) found that 64% of mothers initiated breast-feeding within 24 h of birth. Many studies from India and other South Asian countries have indicated that women commonly wait several days after birth to begin breastfeeding with other foods or liquids. Singh (2002) in a study in Ghaziabad found that 63.9% of mothers started the breast-feeding within 24 h, which is in close approximation to the present study.

According to NFHS-3, UP (2005-06),(10) only 7.3% mothers initiated breast-feeding within 1 h, which is in contrast for our study, while national average of percentage of initiation of breast-feeding was 37.1% within 24 h of birth. Edmond *et al*. (2006)(11) in their study in Ghana found that breast-feeding was initiated within the first day of birth in 71% of infants.

The present study showed that more than one-third (43.5%) of the mothers had given colostrum to their newborn baby. In contrast to our study, Singh (2002) had shown in his study that about 47.8% had given colostrum to the neonate which was approximately similar to our study. NFHS-2, UP (1998-99), reported that only 24.4% of mothers had given colostrum to their babies which less than in the present study. In contrast to the present study, Taja *et al*. (2001)(12) in a district of MP found that 77.3% mothers discarded colostrum and only 22.7% of mothers had given it to their baby.

In present study of the out of those who had not given colostrum, 66.9% not given because they think that it is harmful for their baby and 14.5% of mothers not given colostrum due to ignorance about advantages of this and same percentage of mothers had not given it due to prohibition of elderly female. In contrast to our study, Singh (2002) showed in his study that about 36.8% mothers gave no milk secretion as the reason for not giving colostrum, 28.9% said that they did not know that it should be given, while 18.4% said that giving colostrum was against the tradition of the family and community another 15.7% said that elderly female prevented them from giving colostrum.

## Conclusion

In majority of cases, correct knowledge and correct practices regarding newborn care were lacking among mothers and this should be promoted through improved coverage with existing health services.
